# Different Approaches to Meaning in Primate Gestural and Vocal Communication

**DOI:** 10.3389/fpsyg.2018.00478

**Published:** 2018-04-05

**Authors:** Katja Liebal, Linda Oña

**Affiliations:** Comparative Developmental Psychology, Department of Education and Psychology, Freie Universität Berlin, Berlin, Germany

**Keywords:** meaning, vocalization, gesture, modality, referential, intentional, primates, human language

## Abstract

In searching for the roots of human language, comparative researchers investigate whether precursors to language are already present in our closest relatives, the non-human primates. As the majority of studies into primates’ communication use a unimodal approach with focus on one signal type only, researchers investigate very different aspects depending on whether they are interested in vocal, gestural, or facial communication. Here, we focus on two signal types and discuss how meaning is created in the gestural (visual, tactile/auditory) as compared to the vocal modality in non-human primates, to highlight the different research foci across these modalities. First, we briefly describe the defining features of meaning in human language and introduce some debates concerning meaning in non-human communication. Second, with focus on these features, we summarize the current evidence for meaningful communication in gestural as compared to vocal communication and demonstrate that meaning is operationalized very differently by researchers in these two fields. As a result, it is currently not possible to generalize findings across these modalities. Rather than arguing for or against the occurrence of semantic communication in non-human primates, we aim at pointing to gaps of knowledge in studying meaning in our closest relatives, and these gaps might be closed.

## Introduction

Human language is characterized by a number of ‘design features’ (Hockett, 1960). *Semanticity*, with signals linked to specific meanings, is closely related to other design features. For example, *arbitrariness* refers to the lack of a natural connection between the signal’s signifying form and its signified meaning – the concept to which it refers (de Saussure, 2003/1916). *Duality of patterning* represents the ability to combine a limited set of meaningless components (phonemes) into meaningful structures (morphemes, words) and even longer, more complex sequences (sentences), organized based on specific rules (syntax), while *productivity* refers to the capacity to produce an infinite number of expressions. Since the purpose of such signals is communication, intentional use (*specialization*) is another key feature of language (Hockett, 1960). Although there are various other features characterizing human language, we will focus on this selection, as these features are closely linked to meaning in human language. They are therefore central to our comparative approach to meaning in primate communication.

As comparative researchers assume a gradual evolution of human language, they suggest that precursors to these characterizing features of language are already present in our closest relatives, the non-human primates (hereafter: primates). Consequently, research into their communicative abilities has been shaped by terms and concepts used to characterize human language. It has, however, been questioned whether the use of linguistic terms is justified when describing the communicative abilities of primates (Scott-Phillips, 2008). For example, regarding meaning, Rendall et al. (2009) argue that animal signals merely influence others, but they cannot convey semantic information (but see Scarantino, 2010). While in human language, meaning “…is the central explanatory construct …[which] arises from the common representational states of speakers and listeners” (Rendall et al., 2009, p. 234), in primates, signalers neither intend to inform others when communicating nor do they consider whether recipients need this information (but see Crockford et al., 2012). Meaningful communication, however, involves “overt intentionality”, which requires the sender to “…produce signals with an intention that receivers recognise that the signaller has such intention” (Scott-Phillips, 2016, p. 233). This intentional structure is suggested to be an inherent component of meaningful communication (Grice, 1957), fueling the debate whether such ostensive communication – with the signalers’ communicative intention accompanied by their intention to inform – is unique to humans (Scott-Phillips, 2015b) or shared with other primates (Moore, 2016).

Here, we will not argue in favor of or against claims for meaningful communication in other primates. Rather, our aim is to demonstrate that in research on primate communication, meaning is conceptualized very differently depending on the signal type studied. We will contrast research into gestural communication – including visual, but also tactile and auditory gestures – and vocalizations of primates with regard to those features relevant for meaning: the roles of the sender and the recipient for identifying meaning, the relationship between the signal and its reference (*arbitrariness*), intentional use (*specialization*), and the combination of different signals into meaningful sequences (*duality of patterning, productivity*). Note that as some of these features are closely related or might partly overlap, it is not always possible to address each of them separately.

Primates communicate via different sensory channels, including olfactory, tactile, visual and auditory signals. However, signals are often not differentiated based on their sensory modality, but are rather categorized based on the different cognitive mechanisms assumed to underlie their usage (Liebal et al., 2013b). Consequently, researchers distinguish gestures, facial expressions, and vocalizations. Here, we focus on gestures and vocalizations only, as there are virtually no studies on meaningful combinations or intentional usage of facial expressions (for exceptions, see Waller et al., 2015; Scheider et al., 2016).

Gestures are movements of the limbs or head (e.g., ‘extend arm’, ‘head shake’) or body postures (e.g., ‘present’). As they can comprise different sensory modalities, visual gestures (e.g., ‘wrist offer’) are differentiated from tactile (e.g., ‘slap’) and auditory gestures (e.g., ‘chest beat’), with the latter producing a sound, which does not engage the vocal folds (Call and Tomasello, 2007). Vocalizations (or calls), however, emerge from the larynx, unlike sounds (e.g., ‘raspberries’, ‘whistles’), which do not engage the focal folds.

## Relationship(s) Between Signals and Their Referents (*Arbitrariness*)

Whether signals have meaning(s) is closely linked to whether they refer to specific referents. In human communication, the exact nature of the relationship(s) between a signal and its referent may vary, as reference is differently conceptualized across disciplines and modalities (Leavens et al., 2005). While linguists use “reference” synonymously with symbolic reference to indicate that in spoken language, “a word stands for something”, developmental psychologists also consider non-verbal means of communication in the form of referential gestures, such as pointing gestures of pre-linguistic children (Iverson and Goldin-Meadow, 2005), which can be used to refer to different referents. Furthermore, while linguists highlight the arbitrary relationship between a word and its referent, developmental psychologists suggest that for pointing gestures, the triadic relationship between signaler, recipient, and the external entity is not arbitrary, as “…a point’s specific meaning is determined in large part by the spatial locations of the pointer, the thing indicated, and the communicative partner” (Leavens et al., 2005, p. 185). Together this shows that in human communication, reference is treated differently in spoken language compared to visual non-verbal communication.

Likewise, for primates, comparative researchers operationalize reference differently depending on signal type. Vocal researchers focus on context-specific vocalizations to find “…the animal equivalent to referential words in human language” (Liebal et al., 2013b, p. 399), in the form of functionally referential vocalizations. They are produced in response to a specific stimulus (the referent, e.g., a predator), with receivers showing a specific response to these calls, even in the absence of the eliciting stimulus, indicating that this response itself is stimulus-independent (Macedonia and Evans, 1993; Evans, 1997). As it is unclear whether primates’ calls refer to a specific referent, for example, a predator (“leopard”), or are requesting a specific action in response to this referent (“go up tree”), the term “functional” is used for primates’ referential vocalizations to distinguish them from human referential communication. The ground-breaking finding that vervet monkeys use distinct predator-specific alarm calls in encounters with their main predators (eagles, leopards, pythons) sparked great interest in such functionally referential vocalizations, as playback experiments confirmed that the monkeys showed predator-specific responses upon hearing the corresponding alarm call (Seyfarth et al., 1980). Although claims suggesting the “word-like” nature of these alarm calls have not been confirmed, many following studies found evidence for functionally referential vocalizations in many primate species. They vary in their degree of specificity as they may refer to specific (e.g., leopard versus eagle) or types of predators (e.g., aerial or terrestrial) (Schel et al., 2009), and have been found in different contexts, such as predation, feeding, or social behavior (Di Bitetti, 2003; Slocombe et al., 2010).

In the gestural modality, visual signals in the form of pointing gestures have received considerable attention regarding their referential function. In humans, pointing gestures emerge early in ontogeny (Liszkowski et al., 2004), and are used to refer to different external entities, such as objects, persons, or events. Thus, pointing gestures have no one-to-one referential meaning; instead, the meaning of a pointing gesture depends on its context of use and the common ground shared by the gesturer and the recipient (Liebal et al., 2013a). In primates, the use of pointing gestures has mostly been studied in interactions with humans (Call and Tomasello, 1994; Leavens et al., 1996; but see Vea and Sabater-Pi, 1998; Hobaiter et al., 2014), within which they use these gestures to request food rewards or objects they cannot obtain otherwise (Bullinger et al., 2011). Like in humans, the meaning of primates’ points depends on the context and the common ground primates share with the human experimenter (Bohn et al., 2016). Iconic gestures represent another type of referential gestures, which depict specific objects or actions, resulting in a non-arbitrary relationship between the gesture and the referent. Although concepts of iconicity differ across studies (Perlman et al., 2014), there is some evidence that primates use iconic gestures, mostly to request specific actions (Tanner and Byrne, 1996; Douglas and Moscovice, 2015).

Together this shows that the nature of relationships between a signal and its referent(s) varies across modalities: while some vocalizations are functionally referential signals that refer to specific referents, the relationship between a gesture and its referent(s) varies across gesture types. Pointing gestures can be used to refer to different entities, while iconic gestures depict specific actions.

## Intended Versus Extracted Meaning: the Roles of Signalers and Recipients (*Semanticity* and *Specialization*)

Inspired by ethology, some scholars suggest differentiating between the “messages” of the signaler and the “meaning” extracted by the receiver (Smith, 1965; Font and Carazo, 2010). Meaning is thus conceptualized very differently depending on whether the focus is on the signaler’s or recipient’s behavior. Vocal studies traditionally focus on the recipient. By using playback studies, researchers investigate recipients’ responses toward specific vocalizations to extract their meaning, while they consider contextual information or the signaler’s behavior to a much lesser extent than gesture studies. In the gestural domain, it is not possible to use similar playback experiments to elicit responses to specific gestures at least in interactions between conspecifics. Therefore, unlike in vocal communication, gesture researchers focus on the signaler and investigate whether they produce their gestures intentionally. The term “intentional” is applied in a sense that an individual communicates in a purposeful, goal-directed way, by means of voluntarily controlled actions, while this does not necessarily imply that the recipient understands a signaler’s gesture as an intended act of communication. It is also debated whether apes who gesture intentionally could additionally be said to act with communicative intentions (Scott-Phillips, 2015a; Moore, 2016; Townsend et al., 2017). Furthermore, unlike in vocal studies, gesture research largely ignores context-specific signals, as flexible usage is an important criterion to identify intentional communication. Therefore, researchers focus on those gestures used across different contexts and argue that the meaning of a gesture might differ depending on the context in which it is used (Call and Tomasello, 2007). However, although contextual information contributes to identifying a gesture’s meaning, “context” should not be used as a substitute for “meaning”. More recently, gesture researchers have started to also consider recipients’ responses to identify the signalers’ intended meaning when performing a gesture (more in section “New Developments and the Way Forward in Studying Meaning in Primate Communication”), which is more in keeping with vocal research. Importantly, note that “intentional gesture production” has to be distinguished from the “signaler’s intended meaning”: A message is only taken to have an intended meaning (as distinct from an intended effect) if it was produced not only intentionally, but with communicative intent – that is, if it was produced both intentionally and ostensively (Scott-Phillips, 2015a; Moore, 2016).

Unlike in the gestural modality, intentionality in vocal production has received little attention. Vocalizations have been suggested to be involuntary expressions of emotional states (Tomasello, 2008), supported by neurobiological studies indicating that vocal production is largely mediated by several motor nuclei in the pons and the reticular formation in the medulla, with no direct connections to cortical motor areas (Jürgens, 2002). This traditional notion, however, is increasingly being challenged, as it has been shown that several cortical areas (e.g., anterior cingulate gyrus and ventrolateral prefrontal cortex) are involved in the production of volitional calls (Gavrilov et al., 2017). Furthermore, as vocal researchers have started to consider the signaler’s behavior, they found that chimpanzees’ alarm calls are most likely intentionally produced signals (Schel et al., 2013). Chimpanzees even seem to consider conspecifics’ knowledge states, as they only vocalize when unknowledgeable individuals are close to a hidden predator (Crockford et al., 2012).

Thus, to determine a signal’s meaning, gesture researchers usually focus on the signaler’s behavior, while vocal researchers consider the recipient’s reactions. However, research on both modalities is increasingly investigating both signalers’ and recipients’ behaviors to extract the meaning of vocal and gestural signals.

## Creation of New Meanings (*Duality of Patterning, Productivity, Syntax*)

Duality of patterning and productivity are two design features of human language, which both relate to creating new meaningful utterances from an existing, potentially limited repertoire. Comparative researchers are therefore interested in whether primates also combine their signals into meaningful sequences. They investigate whether combinations of several signals are used for different functions than the components they consist of, or alternatively, whether the meaning of one of the components is modified by the other component. Combining several signals is closely linked to the question of whether a specific order is crucial for the creation of new meaning and thus whether such combinations are based on specific syntactical rules.

Zuberbühler (2002) demonstrated that in some situations, Campbell’s monkeys combine their alarm calls with a preceding boom-call, which modifies the meaning of the following alarm call. Thus, while the functionally referential alarm call is uttered in encounters with their predators, they use this specific call combination in less dangerous situations, such as falling trees. Proceeding from this finding, later studies concluded that “…the Campbell’s monkey call system may be the most complex example of ‘proto-syntax’ in animal communication known to date” (Ouattara et al., 2009). A different system was found in Putty-nosed monkeys that use two alarm calls, which are not predator-specific. Interestingly, the reference to specific predators is achieved by producing sequences of calls, as hack-sequences are more likely to be used in response to eagles, while pyow-sequences occur in response to leopards (Arnold and Zuberbühler, 2006). Combinations of the two call types, however, are used to initiate group travels, indicating that by combining these different vocalizations, new meaning is created.

In the gestural domain, sequences are defined as multiple gestures produced one after the other by one individual, toward the same recipient and the same goal, with sequences varying in the number of gestures combined (Liebal et al., 2004). Although findings across species and studies differ, common conclusion are that gestures are not combined in ways to create new meanings and that gesture combinations are not governed by specific rules (e.g., Genty and Byrne, 2010; Roberts et al., 2013; Hobaiter et al., 2014).

This suggests that gesture combinations are not based on combinatorial rules and are not used for different functions than their single components like it has been shown for vocalizations. However, the finding that primates are able to combine vocalizations into more complex sequences with specific meanings is also debated, as “…there is no evidence of the compositionality essential to language—having a few sequences with a well-defined meaning does not qualify as syntax” (Arbib et al., 2008).

## New Developments and the Way Forward in Studying Meaning in Primate Communication

We have shown that meaning in primates is conceptualized and studied very differently in the gestural as compared to the vocal modality (**Table 1**). Rather than representing fundamental differences across modalities, this may reflect different research traditions and historical limitations in methodological approaches. While gesture research focuses on signalers and whether they communicate intentionally, vocal researchers study the recipients’ responses to identify the meaning they extract from a call. Gesture researchers highlight the importance of the context in which an interaction takes place, as it contributes to a gesture’s meaning. They focus on flexible gesture usage as an important characteristic of intentional communication, and are less interested in context-specific gestures. Vocal researchers, however, traditionally focus on context-specific, functionally referential vocalizations. Slocombe et al. (2011) further demonstrated that gestures are usually studied in great apes, in captive settings, by using observational methods, while most research on vocalizations is conducted with monkey species, in their natural habitats, by using experimental methods. These fundamental differences in how meaning is studied across modalities hinder comparisons across signal types and make it difficult to conclude whether there is evidence for meaningful communication in primates. Furthermore, it seems that researchers are often not aware that they use the term meaning very differently, which in turn does not support a fruitful discourse about how comparative approaches contribute to our understanding of language evolution (Bar-On and Moore, 2017).

**Table 1:** Different approaches to studying meaning in primates’ gestural and vocal communication.

	Gestures	Vocalizations
Who is studied?	Signaler (and recipient)	Recipient
Is intentionality considered?	Yes (production)	No
How is meaning studied?	Intended meaning	Extracted meaning
Which signals are studied?	Context-unspecific	Context-specific
What is the relationship between signal and referent?	No one-to-one referential meaning between pointing gestures and their varying referents	One-to-one referential meaning between functionally referential signals and their referents
Which species are studied?	Apes	Monkeys
Which methods are used?	Observations	Playback experiments
Where are studies conducted?	Captivity	Natural habitats


However, in both vocal and gestural research, traditional approaches have been questioned and new approaches for studying meaning have been suggested. For example, in the vocal modality, the concept of functionally referential vocalizations has been recently criticized for a number of different reasons (see Wheeler and Fischer, 2012; Fischer and Price, 2016). First, because of the strong focus on context-specific vocalizations, the prevalence and significance of functionally referential vocalizations might have been overestimated as compared to other, less context-specific vocalizations. Second, it is often assumed that these vocalizations might require more sophisticated cognitive skills than other vocalizations or other signal types, since the differentiated responses of receivers of such calls “…have been widely interpreted as evidence that signals elicit mental representations in receivers based on the information extracted from the signal” (Wheeler and Fischer, 2012, p. 199). However, such specific responses may be explained by lower-level mechanisms such as classical conditioning, “…without drawing on the concept of information, the meaning of calls, or mental representations of a signal’s purported referent in listeners” (Wheeler and Fischer, 2012, p. 199). Because of this, the relationship between a vocal signal and its referent might not be as arbitrary as previously suggested. Wheeler and Fischer (2012) therefore suggested abandoning the concept of functionally referential vocalizations. Rather, meaning in primate vocal communication should be studied in the framework of pragmatics to investigate how primates use contextual information – in addition to the information provided by the signal itself (Wheeler and Fischer, 2012).

In the gestural domain, we can observe the opposite trend. While gesture researchers have previously proposed that gestures do not have inherent meaning, but have rather highlighted the importance of the context for defining the meaning of a gesture (Call and Tomasello, 2007), recent studies emphasize that gestures indeed have specific meaning (Cartmill and Byrne, 2010; Hobaiter and Byrne, 2014; Graham et al., 2018). This new approach focuses on both the signaler and the recipient by investigating if the signaler’s intended meaning when using a gesture matches with a particular outcome (Byrne et al., 2017). If the recipient’s response satisfied the signaler – evident in the signaler stopping the production of a certain gesture – this is referred to as the “apparently satisfactory outcome” of this specific gesture. In other words, the matching of the intended and extracted meaning is used as an approximation of the gesture’s meaning. Hobaiter and Byrne (2014) found that wild chimpanzees use at least some gestures with tight meaning, in a sense that the same outcome was observed in more than 70% of their use, while other gestures have loose meaning, as they elicited the same outcome in only 50–70%. Note, however, that chimpanzees still used the majority of their gestures for multiple outcomes (Hobaiter and Byrne, 2014), as found by other studies, which focused on the flexibility of gesture use, and which therefore concluded that chimpanzee gestures have no inherent meaning, as the meaning is defined by the context in which they are used (Tomasello et al., 1994). This shows that conclusions drawn from such studies depend at least partly on which findings are emphasized: while some authors focus on those gestures flexibly used across different contexts and conclude that gestures have no meaning, others focus on context-specific gestures, used for one or few outcomes, and consequently emphasize their specific meaning(s). Future research should bring together these two perspectives and study context-specific and unspecific gestures in concert, as gesture types may vary in their degree of specificity, as found for vocalizations. For example, chimpanzees’ visual gestures are more likely to occur in a specific context (e.g., sexual behavior, requesting food) and thus represent “intention movements”, which are abbreviations of full-fledged behaviors used for a specific purpose (Tomasello et al., 1989), while tactile and auditory gestures are often produced across different contexts to trigger others’ actions (Liebal and Call, 2012).

Related to this, it is important to discuss how specific meaning has to be, particularly if we aim at comparing meaning across modalities (Scarantino, 2013). Thus, we have shown that pointing gestures have no one-to-one referential meaning, as pointing can be used to refer to different entities, while functionally referential vocalizations often refer to specific referents. Furthermore, researchers differentiate between tight and loose meanings of chimpanzee gestures, and even gestures with tight meanings may result in multiple outcomes. This highlights the lack of definitions applicable across modalities as well as a lack of a measure based on which the specificity of meaning of a signal can be judged.

Sievers and Gruber (2016) therefore suggest using a “pragmatic notion of reference” that focuses on the use of a signal to refer to something in a specific situation – rather than expecting that signals have referential meaning in themselves. They further highlight that in human language, reference is an action of the signaler and claim that “…any definition describing reference in non-human animals must also focus on the producer” (Sievers and Gruber, 2016, p. 759). In other words, a signal only has referential meaning if the signaler intends to refer to a specific referent. This has important implications, as functionally referential vocalizations have been almost exclusively studied with focus on the recipient. Therefore, to be able to conclude that vocalizations are indeed meaningful, we would have to additionally demonstrate that they are intentionally produced.

Finally, we have argued that unlike in the vocal modality, there is currently no evidence for meaningful combinations in gesture sequences. This may be partly explained by the fact that there is only little research investigating if single gestures are meaningful units. As a result, we are currently lacking sufficient datasets to determine whether a gesture’s meaning changes when it is part of a sequence compared to when it is used in isolation. Furthermore, we want to highlight the importance of multimodal approaches (Slocombe et al., 2010), as it is currently unclear whether combinations consisting of different signal types, such as gesture plus facial expression or gesture plus vocalization result in the creation of new meaning or the modification of an existing one (Hobaiter et al., 2017; Wilke et al., 2017). To study meaningful communication in primates in more comprehensive ways, the essential first step is to combine research efforts across modalities, based on shared definitions which are applicable across signal types and to use a multi-perspective approach, which considers the behavior of both signalers and recipients, in addition to the context.

## Author Contributions

KL wrote the first draft of this manuscript. Both authors then finished the manuscript together and circulated it several times until the current version was finalized.

## Conflict of Interest Statement

The authors declare that the research was conducted in the absence of any commercial or financial relationships that could be construed as a potential conflict of interest.
